# Expanded Heisenberg Hamiltonians from a Mn/Bi DFT+U study on hexagonal antiferromagnet CaMn_2_Bi_2_: excitations and strain-controlled magnetic anisotropy switching

**DOI:** 10.1038/s41598-026-39215-x

**Published:** 2026-03-27

**Authors:** R. H. Aguilera-del-Toro, M. Arruabarrena, A. Leonardo, Martin Rodriguez-Vega, Gregory A. Fiete, A. Ayuela

**Affiliations:** 1 Departamento de Física Teórica, Atómica y Óptica, Universidad de Valladolid, Paseo de Belén, 7, 47011 Valladolid, Castilla y León Spain; 2 Centro de Física de Materiales-Materials Physics Center (CFM-MPC), CSIC-UPV/EHU, Paseo Manuel de Lardizabal, 5, 20018 Donostia, Basque Country Spain; 3 Donostia International Physics Center (DIPC), Paseo Manuel de Lardizabal, 4, 20018 Donostia, Basque Country Spain; 4 EHU Quantum Center, University of the Basque Country UPV/EHU, Barrio de Sarriena, 48940 Leioa, Basque Country Spain; 5 American Physical Society, 1 Physics Ellipse Dr, College Park, MD 20740 USA; 6 Department of Physics, Northeastern University, Boston, MA 02115 USA; 7 Quantum Materials and Sensing Institute, Northeastern University, Burlington, MA 01803 USA; 8 Department of Physics, Massachusetts Institute of Technology, Cambridge, MA 02139 USA

**Keywords:** Materials science, Physics

## Abstract

The manganese pnictide CaMn$_2$Bi$_2$ exhibits narrow-gap antiferromagnetism with Mn atoms arranged in a puckered honeycomb structure, and is currently a promising candidate for ultra-fast light control of AFM states. In this paper, we perform a detailed study of the magnetic properties of CaMn$_2$Bi$_2$ using density functional theory (DFT) combined with the Hubbard U correction and spin-orbit coupling, which accurately describe the magnetic configurations. In DFT+U approach, we apply an on-site U not only to Mn-3d orbitals but also to Bi-6p ones to improve the description of Mn–Bi hybridization and the small SOC-driven gap. We show that a standard Heisenberg spin model is insufficient to describe these magnetic excitations, and an extended model accurately describes these using local on-site magnetization terms, linked to the Néel vector and inspired by Hubbard-model physics. We further investigate the role of the spin-orbit coupling, and find that the magnetic anisotropy of CaMn$_2$Bi$_2$ shows an easy plane, with the preferred magnetization direction being exchanged between axes in the plane by applying small strain values. This strain-tunable magnetization, driven by the interplay between spin-orbit interactions and lattice distortions, highlights the potential for controlling magnetic states in Mn-pnictides for future applications in spintronic and magneto-optical devices.

## Introduction

Layered transition metal based pnictides have attracted much interest in materials physics due to their rich physics properties, including magnetism, superconductivity, charge density waves, and remarkably high magnetoresistance^1,2^. In particular, compounds having Bi in the layers like BaMn$_2$Bi$_2$ and BaMnBiF have emerged as promising counterparts to Fe-based pnictides^3^, because they have lower band gaps and better metallicity. Furthermore, the manganese-based pnictides in the form of AMn$_2$Pn$_2$ (A = Ca, Sr, Ba; Pn = P, As, Sb, Bi) exhibit distinct properties related to structural changes, with Ca- and Sr-based materials crystallizing in the CaAl$_2$Si$_2$-type trigonal structure (space group P3m1) and Ba-based compounds adopting the ThCr$_2$Si$_2$-type tetragonal structure^4^. Among these, the manganese pnictide CaMn$_2$Bi$_2$, with a honeycomb structure of Mn and a narrow electronic gap, currently is a promising candidate for investigating complex electronic phenomena, such as magnetism and light induced magnetic excitations^5^.

Previous studies identified CaMn$_2$Bi$_2$ as a hybridization gap semiconductor, where interactions between localized Mn-*d* states and more delocalized metallic (likely) Bi-*p* states open a small band gap^6^. This compound exhibits unusual magnetism, such as strong anisotropy^6^, non-monotonic magnetoresistance^7^, AFM transitions^5^, and a small band gap, which implies semiconducting transport properties^8^. The importance of the hybridization of Bi$p-$Mn*d* orbitals in influencing the behavior observed in CaMn$_2$Bi$_2$ was highlighted by electronic structure calculations. In addition, measurements using electrical and Hall resistivity experiments suggest that CaMn$_2$Bi$_2$ acts as an extrinsic narrow-gap semiconductor at low temperatures^8^. This manganese pnictide was observed to show an activation gap of 20 K, while at high temperatures it exhibits properties of a single-band semimetal.

Under pressure, experiments reported that CaMn$_2$Bi$_2$ undergoes transitions and becomes a metal, which could be due to structural, magnetic and electronic instabilities^9^. The presence of spin spirals at high pressures was recently shown, which further increases the magnetic complexity in CaMn$_2$Bi$_2$^10^. Furthermore, when a magnetic field is applied, the compound shows magnetic fluctuations and anisotropy^7^. However, a systematic study of the gap, magnetic ordering and strain-tunable anisotropy for CaMn$_2$Bi$_2$ is still lacking using first principles.

Current interest arises from experiments showing that its antiferromagnetic (AFM) order can be optically controlled^5^. For instance, time-resolved second-harmonic generation (TR-SHG) experiments have shown ultrafast, *non-thermal* reorientation of AFM states. These experiments open a new avenue in opto-magnetism, where light can be used to manipulate antiferromagnetic properties. Note that fm-second lasers on CaMn$_2$Bi$_2$ change the magnetic state in the AFM order in time scales within hundredths of picoseconds, which is already relevant to engineering and paves the way to be useful in devices. We thus aim to understand how exchange interactions, spin-orbit coupling (SOC), and external perturbations, such as strain (even induced by light), change the AFM order and anisotropy in CaMn$_2$Bi$_2$.

In this work, we present a detailed study of the bulk manganese pnictide CaMn$_2$Bi$_2$, using density functional theory with Hubbard correction and spin-orbit coupling. To better capture the correlated and hybridized character of this system, we employ a DFT+U scheme in which a moderate U is applied not only to the Mn-3d orbitals but also to the Bi-6p states. This dual-U treatment provides a more accurate description of the Mn–Bi p–d hybridization and the narrow SOC-driven band gap, offering a consistent foundation for the extended spin Hamiltonian analysis presented below. We then investigate the electronic structure, magnetic ordering, and the magnetic anisotropy under the effects of strain, which allows us to exchange the magnetization direction. We then focus on DFT-based modeling to identify stable AFM configurations in CaMn$_2$Bi$_2$. We study the effects of strain on magnetic anisotropy in order to explore additional mechanisms to control the magnetic order, aside from light^5^. We believe that our findings on the unique properties of CaMn$_2$Bi$_2$ provide valuable insights into the mechanisms for the development of spintronics and magnetolectronic devices, and offer a guide for future experimental investigations.

## Results and discussion

**Fig. 1:**
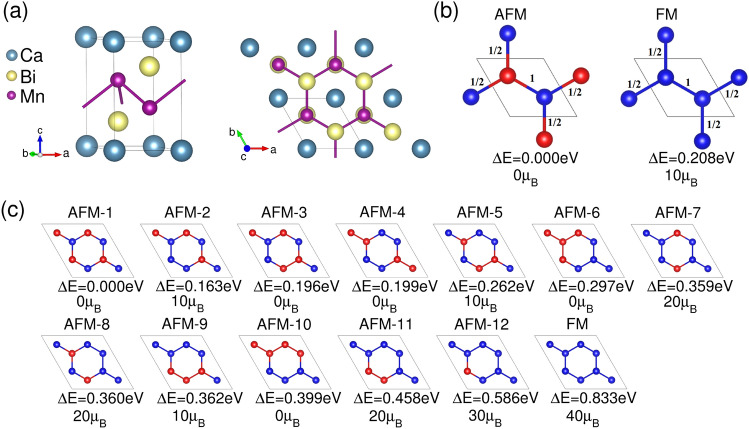
(**a**) Crystalline honeycomb structure of CaMn$_2$Bi$_2$: (left) side view of the unit cell, and (right) top view of the 2 $\times$ 2 $\times$ 1 supercell. The solid black lines are enclosing the considered unit cell. (**b**) Scheme of antiferromagnetic and ferromagnetic configurations in the CaMn$_2$Bi$_2$ unit cell. Red (blue) spheres represent Mn atoms with spin up (down). (**c**) Different magnetic configurations for excitations considering a larger 2 $\times$ 2 $\times$ 1 supercell. The energy differences $\Delta E$ between each configuration the ground state configuration labelled as AFM-1 are given below. The total magnetic moment of each magnetic excitation is also included in $\mu _{\text {B}}$ units.

The crystal structure of CaMn$_2$Bi$_2$ crystal is shown in Fig. 1a. This compound exhibits hexagonal symmetry and is classified in the $P\bar{3}m1$ (164) space group, with the lattice parameters $a=4.64 {{\AA }}$ and $c=7.64 {{\AA }}$, as determined by experimental measurements^8^. The relaxed lattice parameters are $a=4.77 {{\AA }}$ and $c=7.74 {{\AA }}$, in agreement with experiments and those obtained in a recent work using only a $U_{Mn}$ value^11^. This manganese pnictide has an antiferromagnetic ground state, with neighboring Mn atoms in the same layer having opposite spin, as shown schematically in the left panel of Fig. 1b. The structural relaxation and electronic calculations show that the material maintains its layered crystal structure, with Mn and Bi atomic orbitals playing distinct roles in the electronic properties. The Mn states dominate the conduction band, while the Bi states are the primary contributors to the valence band. The results from these calculations provide a solid theoretical framework for understanding the electronic and even possible topological properties of CaMn$_2$Bi$_2$.

**Fig. 2:**
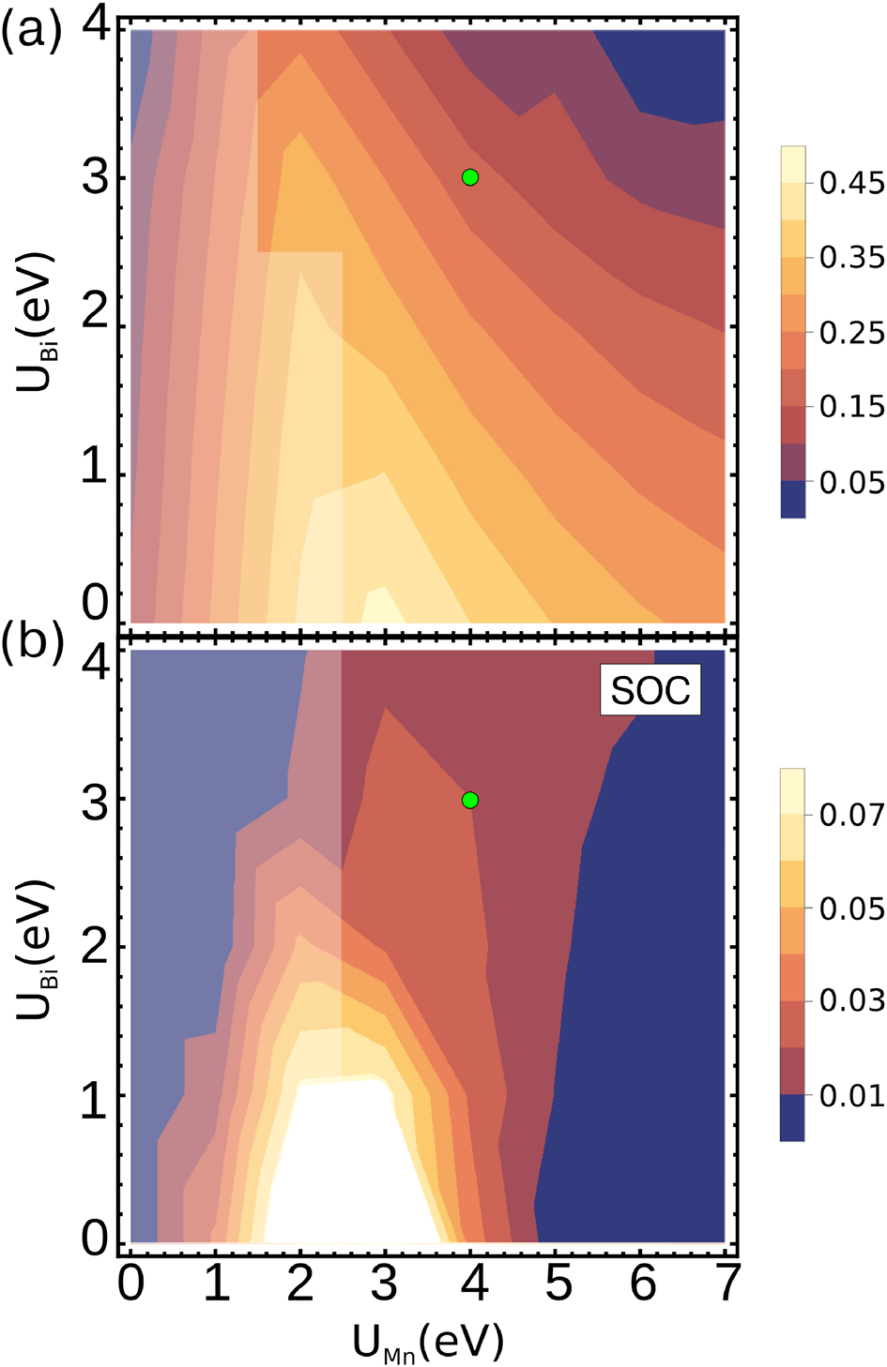
Band gap as a function of the Coulomb parameters $U\,_{\text {Mn}}$ and $U\,_{\text {Bi}}$, (**a**) without spin-orbit coupling, and (**b**) with spin-orbit coupling. The slightly shaded areas depict a region of *U* values with indirect band gaps between the high symmetry $\Gamma$ and M points; another region includes the *U* pairs that show (in)direct band gaps around the $\Gamma$ point. The chosen *U* values $U\,_{\text {Mn}}$= 4eV and $U\,_{\text {Bi}}$= 3 eV used in the calculations in this work are marked with a green circle. Obtained using GGA+U+SOC calculations.

### Electronic properties

#### Spin-orbit coupling and correlations effects on band gap tuning

The electronic properties of CaMn$_2$Bi$_2$ remain a subject of discussion due to discrepancies between theoretical and experimental band gap values. Transport measurements report band gap values between 31 to 62 meV, which classify it as a narrow-gap semiconductor^6^. To compare with experiment, we employed several computational approaches: from standard GGA to GGA+*U* with spin-orbit coupling, as well as the more computationally demanding HSE06 hybrid functional.

*Role of spin-orbit coupling and U parameters on Mn and Bi:* Figure 2 shows the calculated band gap values as a function of $U_{Mn}$ and $U_{Bi}$, both without (panel a) and with (panel b) spin-orbit coupling. Spin-orbit coupling significantly narrows the band gap by lifting degeneracies in the Bi-derived valence bands that becomes closer to the Mn- conduction bands. This narrowing is evident in the color scale range shown to the right of each panel. We find that the band gaps with SOC decrease by an order of magnitude. In GGA+*U* calculations with $U_{\text {Mn}}=4$ eV and $U_{\text {Bi}}=3$ eV, SOC reduces the band gap from 171 meV to 20 meV - nearly an order of magnitude (A similar nonmonotonic trend is observed in Fig. S3 of Ref.^11^). The general trend indicates that the band gap value increases with $U_{\text {Mn}}$ up to a peak, then decreases. In contrast, increasing $U_{\text {Bi}}$ consistently reduces the band gap. It is also important to note that the nature of the band gap depends on the *U* values. For small values of $U_{\text {Mn}}$, the band gap is indirect, between the $\Gamma$ and M points. For $U_{\text {Mn}}$ values greater than 2.5 eV, the gap is nearly direct around the $\Gamma$ point, with the valence-band maximum shifted slightly towards the Gamma-A direction.

*Hybrid calculations:* The band structure of CaMn$_2$Bi$_2$ with GGA+*U* and spin-orbit is compared to that using the HSE06 hybrid (included as Fig. S1). While the bands comparison shows qualitative agreement, the hybrid calculations significantly overestimate the band gap, a well-known issue in this class of materials^8^. However, we find that the band gap decreases from values of 0.7 eV (hybrid HSE06) to 20 meV (GGA+*U* with SOC).

It is noteworthy that the GGA+ U approach particularly with the selected values $U_{\text {Mn}}=4$ eV and $U_{\text {Bi}}=3$ eV provides a realistic gap value close to experimental transport measurements. These results suggest that GGA+*U* calculations including SOC should be the preferred method in the following for describing CaMn$_2$Bi$_2$ electronic structure, as it offers a reliable and computationally efficient description.

#### Electronic bands and orbital contributions

**Fig. 3:**
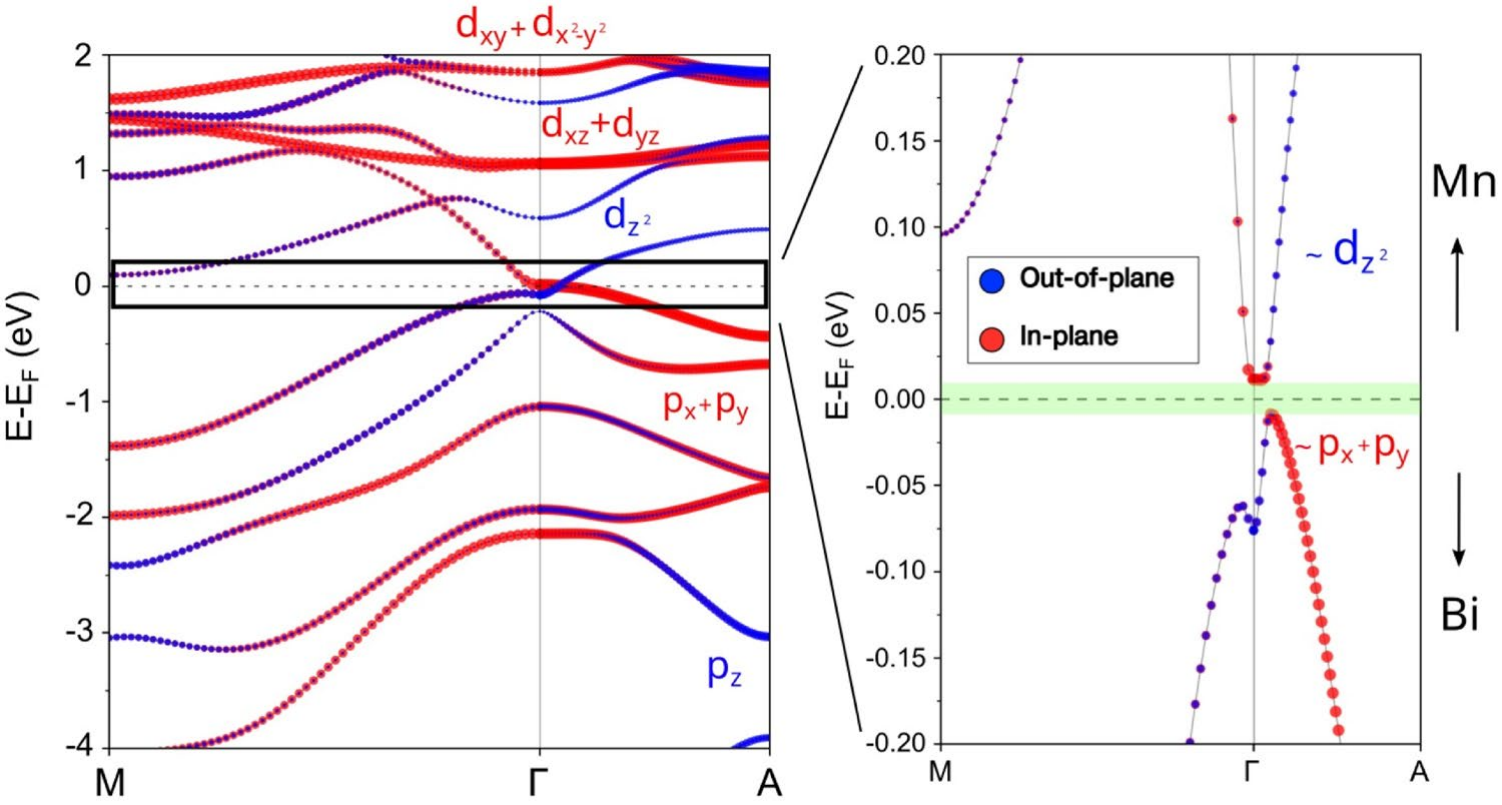
Band structure of CaMn$_2$Bi$_2$ computed using the GGA+*U* approach. In red, the projection into the orbitals with in-plane character is shown (Bi p$_x$, p$_y$ and Mn d$_{xy}$, d$_{x^2-y^2}$, d$_{xz}$, d$_{yz}$ ); while in blue, the out-of-plane projection is displayed (Bi p$_z$ and Mn d$_{z^2}$ ). In the right panel, the region around the Fermi energy is zoomed in, highlighting the 20 meV narrow band gap of this compound. Obtained using GGA+U+SOC calculations.

Figure 3 shows a detailed inspection of the band structure near the Fermi level around the $\Gamma$ point. The band gap is narrow, with a small energy difference between the conduction band minimum and valence band maximum. Understanding the orbital character is essential to explain the band gap behavior and the role of spin-orbit coupling SOC. The analysis of the projected band structure confirms that Bi p orbitals are dominant to the valence states, and Mn d orbitals primarily contribute to the conduction bands, which is consistent with a hybridization-driven origin of the electronic gap. These findings agree with the large modification of the electronic structure due to the computational method, especially the critical role of spin-orbit coupling in shaping the gap and bands, as described below.

We next study the Bi conduction states near the $\Gamma$ point, initially without spin-orbit coupling (given in Fig. S3a). The degeneracy of the states shows in-plane $(p_x,p_y)$ and and out-of-plane $p_z$ Bi orbitals with two- and one-degeneracy, respectively. The couple of bonding-antibonding $p_z$ orbitals are centered around -3.5 eV, and the $(p_x,p_y)$ states appear around -0.5 eV below the Fermi level, which implies a Bi crystal field of approximately 3. eV. The Mn contributions to the $(p_x,p_y)$ and $p_z$ Bi bands vary with the k-point direction. Note that the Mn $d_z^2$ states are more hybridized with the occupied $p_z$ bands near the Fermi energy at the $\Gamma$ point.

The spin-orbit contribution especially from Bi atoms is significant, as shown in the bands of Fig. 3 and their orbital decomposition included in Fig S3b. Including spin-orbit coupling lifts the double degeneracy shown by the $(p_x,p_y)$ states and induces their mixing with out-of-plane orbitals. The calculated value for this splitting is large on the order of a few tenths of an eV. Consequently, spin orbit mixing removes band crossing along the in-plane $\Gamma -M$ and out-of-plane $\Gamma -A$ directions. As a result, the gap becomes smaller in the order of few meVs, as seen in the zoomed-in region of Fig. 3. The gap is like direct around the Gamma point, with the valence-band maximum shifted slightly towards the Gamma–A direction. This SOC-induced band gap reduction to a few meVs, particularly near the $\Gamma$-point, plays a crucial role in enabling strain-tunable magnetic anisotropy and light-driven magnetic control.

It is noteworthy that the gap minimum is slightly shifted from the $\Gamma$ point because of spin-orbit mixing between in- and out-of plane orbitals. Figure 3 also includes the key orbital contributions to the electronic structure of CaMn$_2$Bi$_2$. The valence bands near the Fermi level predominantly originate from Bi $(p_x,p_y)$ orbitals, which hybridize strongly with the Mn $d_{zx}$ and $d_{zy}$ states and produce anisotropic bands in the MnBi plane. In contrast, the conduction band shows a significant contribution from the Mn $d_{z^2}$ states hybridized with Bi $p_{z}$, particularly near the $\Gamma$ point. This hybridization, enhanced by spin–orbit coupling, lifts the $p_{x,y}$ degeneracy and mixes these Bi orbitals not only with Bi $p_z$ but with Mn $d_{z^2}$, so that the conduction-band minimum and valence-band top going from to $\Gamma$ to A have Bi and Mn character, respectively. This mixing is an indication of band inversion effects. The strength of Mn-Bi hybridization varies with the k-direction and indicates a strong anisotropy in Mn-Bi layers, which may not only change the gap and electronic properties but also add potential topological behavior to this pnictide, as seen in related compounds^12^.

### Magnetic properties

#### Magnetic Hamiltonian and excitations

**Fig. 4:**
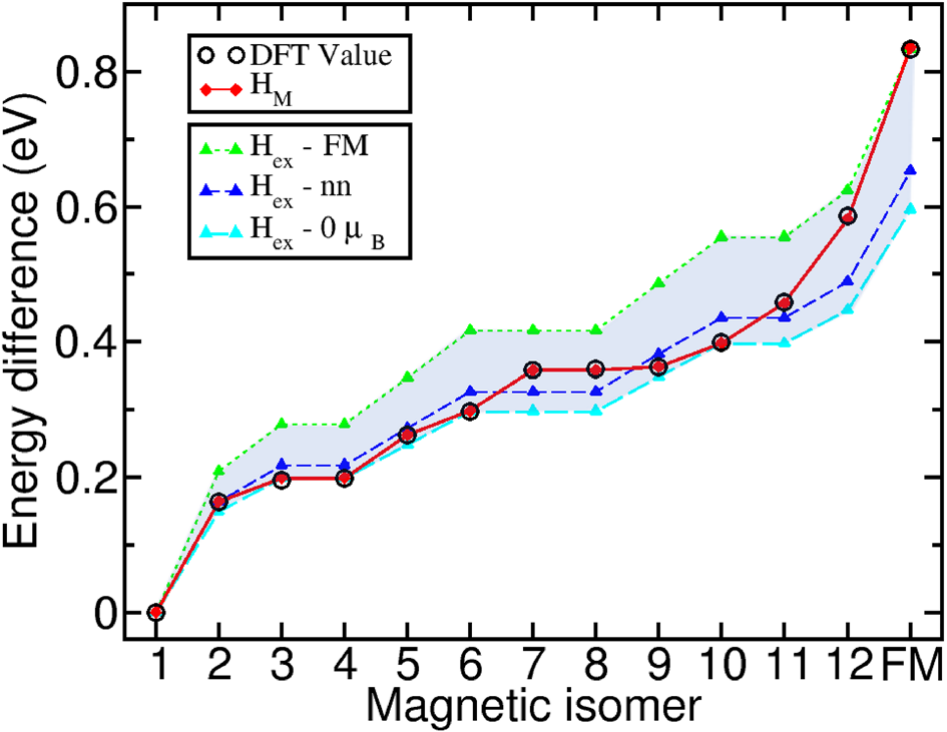
Energy difference between the magnetic excitations in the 2x2x1 supercell and the ground state AFM-1 configuration. The black marks show the energy difference obtained directly by subtracting the DFT total energies. The values in green are calculated using the H$_{\text {ex}}$ model described in Eq. 1. The energy differences in blue color are obtained by fitting with the nearest magnetic excitation (H$_{\text {ex-nn}}$). The values in red are calculated using the H$_{\text {M}}$ model, which depends on the magnetization of the cell (Eq. 5) required to describe properly the magnetic excitations.

To investigate the magnetic order of CaMn$_2$Bi$_2$ we employed a 2x2x1 supercell of the chemical unit cell. Figure 1c shows all the tested magnetic configurations, ordered by their calculated energies relative to the antiferromagnetic (AFM) ground state. The corresponding energy differences for each magnetic configuration are shown in Fig. 4. Our results indicate that the magnetism mainly arises from the spin density localized around the Mn atoms. In our system, the Mn atoms exhibit a nearly half-filled 3d$^{5}$ configuration (nominal Mn$^{2+}$), which naturally leads to a high-spin state with a moment close to $5\,\mu _B$. However, the calculated local Mn moments, about $\pm 4.5$–$4.7\,\mu _B$, consistent with this configuration, show a non-negligible decrease due to the Mn hybridization with Bi *p* states. This hybridization is also evident from the induced moments of about 0.3–$0.4\,\mu _B$ on Bi atoms and $0.05\,\mu _B$ on Ca, which confirm a measurable spin polarization of some non-magnetic sublattices.

The ground state exhibits AFM order between Mn layers, with two mutually antiparallel Mn sublattices within each hexagonal layer.

Since the ground state can be obtained using the chemical unit cell, we investigated how to fit the calculated energies to the Heisenberg spin exchange term. Given the crystal symmetry, the exchange coupling strength (J$_e$) can be assumed to remain constant for all Mn–Mn interactions. We therefore express the Heisenberg Hamiltonian as follows:

1 $$\begin{aligned} \small H_{ex}=\sum _{i,j}^{}J_{e} {\bf {S}}_{i}\cdot {\bf {S}}_{j}. \end{aligned}$$

where $S_i$ and $S_j$ are the localized moments at sites i and j respectively. The total energies of the ferromagnetic (FM) and antiferromagnetic (AFM) configurations shown in Fig. 1 are given by

2 $$\begin{aligned} \small E_{FM}=E_0 + 4(\frac{1}{2}J_{e}S^{2})+J_{e}S^{2}= E_0 + 3J_{e}S^{2}, \end{aligned}$$

3 $$\begin{aligned} \small E_{AFM}=E_0 - 4(\frac{1}{2}J_{e}S^{2})-J_{e}S^{2}= E_0 - 3J_{e}S^{2}, \end{aligned}$$

where $E_0$ represents the total energy for the non-magnetic configuration, and $S=5/2$ corresponds to the spin on each manganese atom. Here, we considered the interaction between the Mn atoms and their nearest neighbors in the adjacent cells, as shown in Fig. 1b. The expression for the exchange coupling (J$_e$) is then:

4 $$\begin{aligned} \small J_{e}=\frac{E_{FM}-E_{AFM}}{6S^2} = -5.5 \text {meV}, \end{aligned}$$

where the negative sign indicates the preference for AFM coupling between the Mn atoms. With this exchange coupling we obtain a Hamiltonian fitted to the FM configurations and labeled as $H_{ex-FM}$. Since this Hamiltonian depends on a single parameter, we add to $H_{ex}$ an extra label *FM* that indicates the magnetic configuration relative to the ground AFM state chosen to fit the exchange coupling. In the following other labels can be used to define other fits to different magnetic excitations.

Next we tested the $H_{ex}$ Heisenberg Hamiltonian to calculate the energy differences of magnetic configurations on a larger 2x2x1 supercell, as shown in Fig. 4. Although the energies of all other magnetic excitations fall between the AFM and FM solutions, the Heisenberg model yields significant errors of $\sim$0.1 eV for most magnetic configurations. To improve accuracy we then refitted the exchange constant $J_e$ using next-nearest neighbors instead of the fully FM configuration. We use the 2x2x1 supercell and fit all the magnetic configuration energies with the next-nearest magnetic excitation, and then solve for a new Hamiltonian, given as H$_{ex-nn}$. We obtained an exchange coupling value of -4.35 meV. Note that fitting $H_{ex}$ is just scaling the sum by a factor to reproduce a value or even a set of values. For instance, we have even tried to fit the solutions with zero magnetization, shown as H$_{ex-0 \mu _B}$, and found that the configurations with non-zero magnetization are still not well described. The error in the fitted energies remains unacceptably large compared to the original GGA+*U* results, being higher than 0.02 eV. This suggests that a Hamiltonian based only on this exchange coupling is not enough to accurately describe the magnetic excitations in CaMn$_2$Bi$_2$.

However, the inclusion of additional terms, such as the anisotropic spin exchange arising from spin-orbit coupling, was not able to explain these energy differences about tenths of eV, as the energies using SOC are much smaller. Further corrections to the Heisenberg Hamiltonian, such as including the antisymmetric exchange (Dzyaloshinskii-Moriya interaction), would also adjust the energies below the order of meV. Consequently, the source of the error must be found in missing terms beyond these conventional spin models.

#### On-site magnetization term and Néel vector

A closer look at the magnetic excitations in the 2 $\times$ 2 $\times$ 1 supercell reveals an interesting trend. We find that the excited magnetic configurations with different total magnetic moments and the same number of exchanged J$_e$ pairs have similar energy differences on the order of hundredths of meV. Thus, this finding suggests that a term related to the total magnetic moment must be included in the Hamiltonian to describe accurately this layered material. It is noteworthy that this term captures the magnetic moment unbalance between the two sublattices and can thus be seen as being related to the Néel vector in antiferromagnets.

To address this, we propose the following Hamiltonian:

5 $$\begin{aligned} \small H_{M}=J_{M}\sum _{i,j}^{} {\bf {S}}_{i}\cdot {\bf {S}}_{j}-\frac{M}{N^2}(\sum _{i}^{} {\bf {S}}_{i})^2, \end{aligned}$$

where J$_{M}$ and M are fitting constants, and N is the number of unit cells in the system. The first term represents the exchange coupling, while the second term corresponds to the square of the total magnetic moment of the supercell. Although this second on-site magnetization term is not standard in conventional spin models (The quadratic term $(\sum _i {\bf {S}}_i)^2$ should not be interpreted as a single-ion anisotropy contribution of the form $\sum _i (S_i^z)^2$. These two terms have fundamentally different physical meanings. The single-ion anisotropy acts locally on each magnetic site and favors specific crystal directions, while being negligible compared to the quadratic term, as shown in the MAE section below. In contrast, the quadratic term depends on the net magnetization of the entire supercell and captures sublattice imbalance—a key feature for describing Néel-vector deviations in AFM systems. In any case, there are other possible Hamiltonians that could be used to fit the DFT data, e.g. including further neighbours, but this would require to consider additional spin terms, a study which is beyond the scope of this paper.), it is not just an overall energy shift. Instead, it captures the energy cost associated with the imbalance between sublattices, effectively acting as a penalty for deviations from the ideal Néel vector in AFM systems. The second term in Eq. 5 can be understood by second-order perturbation theory in $t/U$ within the Hubbard model. In the strong-coupling regime at half-filling, virtual excitations lead to an effective magnetic interaction between localized spins, which are well-described by the Heisenberg model as an effective low-energy Hamiltonian with ${\bf {S}}_i \cdot {\bf {S}}_j$ terms^13^. However, beyond nearest neighbors, higher-order or collective spin contributions introduce additional terms such as $\left( \sum _i {\bf {S}}_i \right) ^2$^14^. These terms effectively arise in various extended or cluster-based treatments of the Hubbard model, and capture long-range spin correlations or sublattice imbalances. Therefore, including this term is not ad hoc, but consistent with the expected form of effective spin Hamiltonians derived from strongly correlated electron systems. It is crucial for quantitatively reproducing the energy hierarchy of magnetic excitations as obtained from our DFT+*U* calculations. Fitting the energy data to this model gives the following values for these constants: J$_{M} = -3.97$ meV and $M = -2.39$ meV.

**Fig. 5:**
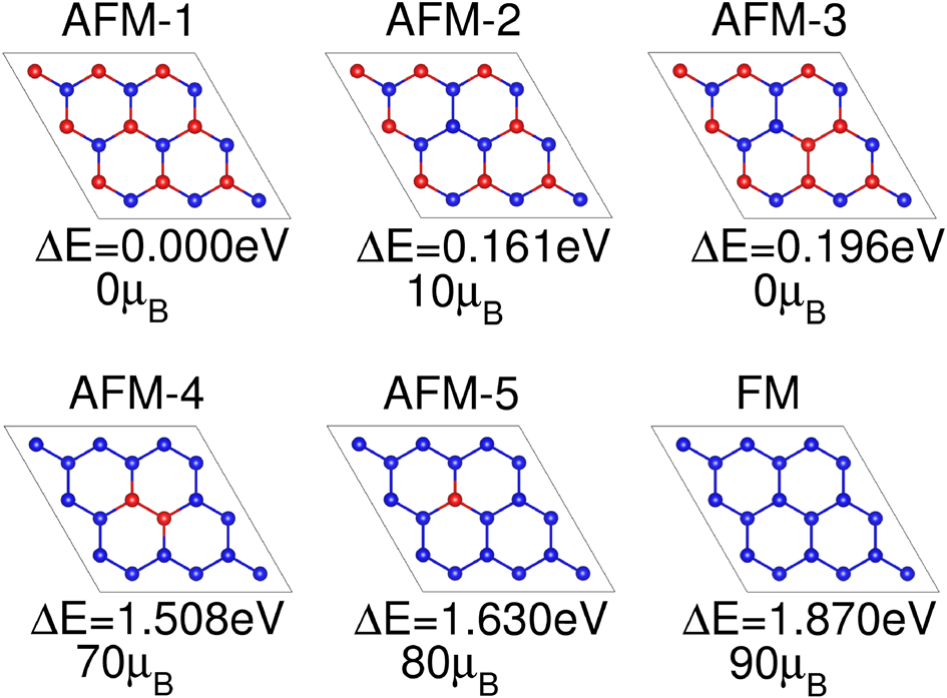
Magnetic excitations for the 3 $\times$ 3 $\times$ 1 cell. Below each case, the energy difference in eV with respect to the ground state and the magnetic moment in $\mu _B$.

In Fig. 4, we compare the energy differences obtained from the GGA+*U* calculations with those predicted using the H$_{ex}$, H$_{ex-nn}$ Hamiltonians, described above, and with the H$_{M}$ one, described here. We observe that the curves considering only the exchange coupling term yield energy values that deviate significantly from the GGA+*U* calculations. However, when using the $H_M$ Hamiltonian model that incorporates the total magnetic moment of the supercell, we can reproduce the energies with errors on the order of tenths of a meV. To validate this model, we also performed calculations on a 3 $\times$ 3 $\times$ 1 supercell (see Fig. 5) and compared the calculated energies with those predicted by the H$_{M}$ Hamiltonian. For this system, the errors falls within the range of hundredths to even thousandths of an electron volt. This suggests that $J_M$ is nearly converged when considering extended local environments. The successful mapping of the $DFT+U$ energy landscape onto the effective spin model ensures that the resulting exchange parameters are already including some quantum state effects. This, coupled with the relatively large Mn spin (below S=5/2), justifies the use of an effective spin model treatment for dealing with the magnetic excitation energies. Thus, this framework means that incorporating local magnetizations is necessary and effectively capture magnetic excitations out of equilibrium- such as those induced by ultrafast laser pulses or high-temperature quenching in the CaMn$_2$Bi$_2$ AFM system.

#### Non-linearities of magnetic anisotropy with strain

Finally, we examine the magnetic anisotropy exhibited by the compound. Including spin-orbit term effectively couples the spin magnetic moment to the crystal lattice, a fact which makes certain magnetic configurations to be either energetically favored or not depending on the orientation of the local Mn magnetic moments. The magnetocrystalline anisotropy energy (MAE) is defined as the energy difference between a given spin orientation and the lowest-energy configuration. We calculated the total energy of CaMn$_2$Bi$_2$ for various spin orientations and found that it predominantly favors an in-plane configuration, with the Mn spins aligned along the hexagonal a-axis. Specifically, the out-of-plane MAE is approximately 3 meV, while the in-plane anisotropy is about 0.02 meV, a difference that highlights the strong easy-plane character of this manganese pnictide. The value of the magnetic anisotropy energy (MAE) in CaMn$_2$Bi$_2$ is hundreds to thousands of times larger than that of traditional magnets (Fe, Co, Ni)^15,16^, whose anisotropy energy is typically in the $\mu \text {eV}$ per atom range.

**Fig. 6:**
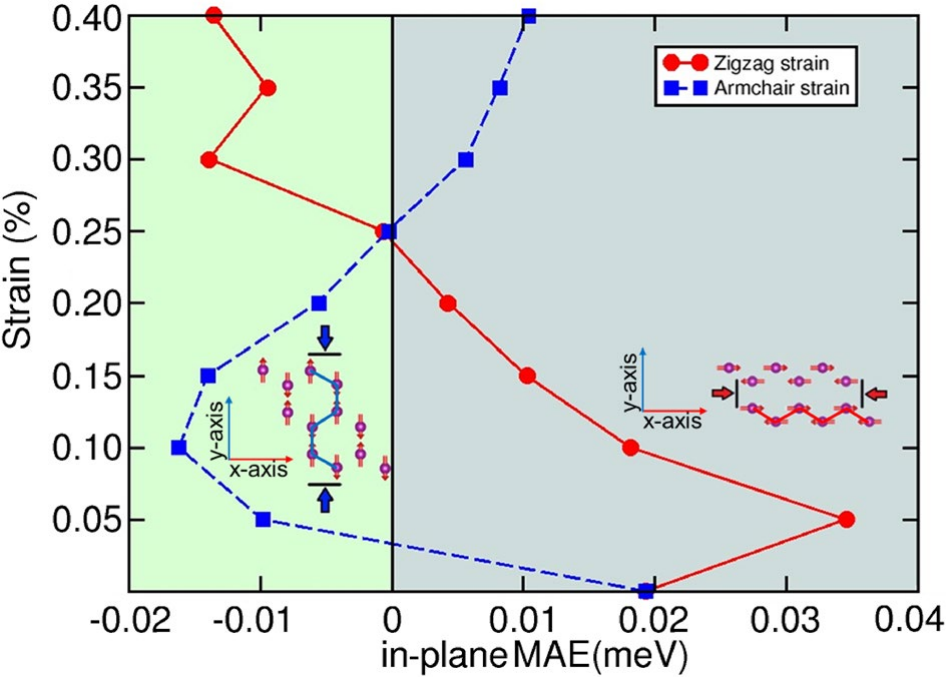
Map of the in-plane magnetic anisotropy energy when the strain is applied along the two main hexagonal directions. The bluish region indicates that the zigzag direction is preferred; the greenish region indicates that the armchair direction is preferred. Obtained using GGA+U+SOC calculations.

We also investigated the effect of strain on magnetic anisotropy. Two uniaxial in-plane strains along the zigzag (*x* axis) and armchair (*y* axis) directions were independently applied in the range from $0.00\%$ to $0.40\%$ in steps of $0.05\%$. Our analysis shows that the in-plane MAE relative to the cartesian *z*-axis (hexagonal *c*-axis) remains nearly constant under strain, while the difference in anisotropy between the in-plane *x* (hexagonal *a*, armchair) and *y* (zigzag) directions changed with strain applied along those axes, as shown in Fig. 6.

Specifically, when strain is applied along the zigzag direction, the preferred spin orientation shifts to the armchair axis at around 0.25% strain. With further strain above 0.4% along the zizzag axis, the anisotropy energy difference increased, and the armchair-axis is comparable to the principal easy axis. Finally, applying strain in the *y*-direction causes the spin orientation to switch from the zigzag to the armchair direction, with an oscillatory behavior up to 0.25% strain, before reverting to the zigzag direction.

Last but not least, the MAE of the AFM ground state exhibits non-linear dependence on strain, whether applied along the zigzag or armchair directions. A key finding from our DFT calculations is that strain change the easy-axis magnetization, effectively reorienting the spin direction. This tunability offers a pathway for mechanical control of AFM order, which may be exploited in strain engineering spintronic applications.

## Methods

### Computational details

The electronic structure calculations for the pnictide structures in this paper were performed using density functional Theory (DFT) as implemented in the Vienna Ab-initio Simulation Package (VASP)^17,18^. The projector augmented wave (PAW) method was employed, and for the exchange and correlation potential we use the Perdew-Burke-Ernzerhof form of the generalized gradient approximation (GGA). Correlation effects in the Mn and Bi states were treated applying DFT+U according to the Dudarev formulation with an effective Coulomb $U_{eff}$ parameter^19^. All electronic calculations used well-converged parameters, including a plane-wave cutoff energy of 700 eV, and a gamma-centered 15x15x8 Monkhorst-Pack k-point mesh. The electronic self-consistency parameter was 10$^{-7}$ eV. The atomic positions were relaxed until the residual forces in all directions were below 0.5 meV/Å.

To account for relativistic effects, we performed fully relativistic calculations including spin-orbit coupling (SOC). We specifically studied the role of SOC on the electronic band structure and the band gap. We also analyzed the orbital character of the states around the band edges to understand the contribution from the Bi and Mn orbitals. As a benchmark, we also calculated the band structures of CaMn$_2$Bi$_2$ using the hybrid HSE06 functional for comparison. Based on a systematic benchmarking to hybrid functional calculations and experimental band gap measurements (see “Computational details”), we select $U_{\text {Mn}}=4$ eV and $U_{\text {Bi}}=3$ eV. A +U correction was also added to the Bi 6p states to account for orbital localization and improve the accuracy of hybridization-gap predictions, which is in line with other works on Bi compounds^20,21^

## Conclusions

In this work, we have investigated the magnetic properties of the layered pnictide CaMn$_2$Bi$_2$ using GGA+*U* with spin-orbit coupling (SOC). We show that the material is a narrow-gap semiconductor with its properties strongly influenced by Bi-Mn hybridization and SOC effects. We find that accurately describing magnetic excitations in this antiferromagnetic compound require a Heisenberg model augmented by an on-site magnetization term related with the Néel vector. Furthermore, our analysis of magnetocrystalline anisotropy under applied strain reveals a non-linear, strain-dependent switching of the in-plane easy axis. This mechanical tunability of magnetic states offers a promising route for strain-engineered control of AFM order, complementary to optical control techniques. Thus, our results position CaMn$_2$Bi$_2$ as a promising antiferromagnetic semiconductor platform in which light and mechanical strain can be used together to achieve ultrafast and reversible magnetic switching, a control that paves the way for advanced spintronic and magneto-optical device applications.

## Data Availability

POSCAR files corresponding to the 13 magnetic configurations analyzed in Fig. 1 of the main text are provided in the supplementary information. The datasets used and/or analysed during the current study are available from the corresponding author upon reasonable request.
